# Plasma Catestatin: A Useful Biomarker for Coronary Collateral Development with Chronic Myocardial Ischemia

**DOI:** 10.1371/journal.pone.0149062

**Published:** 2016-06-15

**Authors:** Weixian Xu, Haiyi Yu, Weihong Li, Wei Gao, Lijun Guo, Guisong Wang

**Affiliations:** Department of Cardiology, Peking University Third Hospital, Key Laboratory of Cardiovascular Molecular Biology and Regulatory Peptides, Ministry of Health, Key Laboratory of Molecular Cardiovascular Sciences, Ministry of Education, Beijing, 100191, China; Centro Cardiologico Monzino, ITALY

## Abstract

**Backgrounds:**

Catestatin is an endogenous multifunctional neuroendocrinepeptide. Recently, catestatin was discovered as a novel angiogenic cytokine. The study was to investigate the associations between endogenous catestatin and coronary collateral development among the patients with chronic myocardial ischemia.

**Methods:**

Thirty-eight patients with coronary artery chronic total occlusions (CTO) (CTO group) and 38 patients with normal coronary arteries (normal group) were enrolled in the series. Among the patients with CTO, coronary collateral development was graded according to the Rentrop score method. Rentrop score 0–1 collateral development was regarded as poor collateral group and 2–3 collateral development was regarded as good collateral group. Plasma catestatin level and vascular endothelial growth factor (VEGF) were measured by ELISA kits.

**Results:**

The plasma catestatin levels in CTO group were significantly higher than that in normal group (1.97±1.01 vs 1.36±0.97ng/ml, p = 0.009). In the CTO group, the patients with good collateral development had significantly higher catestatin and VEGF levels than those with poor collateral development (2.36±0.73 vs 1.61±1.12 ng/ml, p = 0.018; 425.23±140.10 vs 238.48±101.00pg/mL, p<0.001). There is a positive correlation between plasma catestatin levels and Rentrop scores (r = 0.40, p = 0.013) among the patients with CTO. However, there is no correlations between plasma catestatin levels and VEGF (r = -0.06, p = 0.744). In the multiple linear regression models, plasma catestatin level was one of the independent factors of coronary collateral development after adjustment for confounders.

**Conclusions:**

Plasma catestatin was associated with coronary collateral developments. It may be a useful biomarker for coronary collateral development and potential target for therapeutic angiogenesis in patients with CTO.

## Introduction

Catestatin is an endogenous multifunctional neuroendocrinepeptide. It is one of chromogranin A (CgA)-derived fragments through proteolytic processing. Catestatin was well discovered to inhibit catecholamine release from both chromaffin cells and noradrenergic neurons in a reversible and non-competitive fashion [1]. It can also stimulate histamine excretion from mast cells [2], induce monocyte chemotaxis [3], as well as provide antimicrobial protection [4]. It also acts as an anti-endothelin-1and pro-nitric oxide agent [5], and as a potent vasodilator [2]. Therefore, it plays an important roles in neuroendocrine tumors, or in inflammatory and cardiovascular diseases [6–7].

Previous studies have been revealed that catestatin is closely related to many cardiovascular diseases [8]. The plasma concentration of catestatin is diminished not only in established cases of essential hypertension, but also in the still-normotensive offspring of patients with hypertension [9–10]. Furthermore, catestatin was reported to exert antihypertensive effects in animal studies [11–13]. Plasma catestatin levels had a dynamic changes during acute coronary syndrome [14–15]. Plasma catestatin was not only correlated with left ventricular ejection fraction (LVEF) [16] and malignant arrhythmia in acute AMI stage [17], but also correlated with left ventricular remodeling three months later [16] and the severity of heart failure [18].

Recently, catestatin was discovered as a novel angiogenic cytokine [19]. Angiogenesis is an important process to form new vessels from the pre-existing vasculature in many physiological conditions including embryonic development and wound healing. Angiogenesis is regulated by a complex interplay of antiangiogenic and proangiogenic factors. Defects in the regulation of angiogenesis often result in pathological conditions such as inflammatory diseases, ischemic heart disease, peripheral vascular diseases, proliferative retinopathy, and solid tumors [20].

Theurl M et al. [19] reported that catestatin acts as proangiogenic factor via a basic fibroblast growth factor-dependent mechanism. In his study, in vitro, in endothelial cells, catestatin induced migration, proliferation and exerted capillary tube formation via G protein, mitogen-activated protein kinase. Catestatin released basic fibroblast growth factor from endothelial cells and stimulated fibroblast growth factor signaling resulting in angiogenesis. In vivo, in the mouse cornea neovascularization assay, catestatin induced angiogenesis and increased blood perfusion and number of capillaries; in the hindlimb ischemia model, catestatin increased density of arterioles/arteries and incorporation of endothelial progenitor cells indicating induction of arteriogenesis and postnatal vasculogenesis, besides angiogenesis [19].

Coronary artery disease (CAD) results in myocardial ischemia and its manifestations include acute myocardial infarction and angina. Angiogenesis inducing collateral development is one of the important compensatory mechanisms to myocardial ischemia, especially in the setting of coronary artery chronic total occlusions (CTO), because it can provide an extra blood supply and salvage the myocardium in the ischemic region. The degree of coronary collateral development showed a significant variation among patients with coronary artery disease. The extent of angiogenesis was thought to be related to the degree of myocardial ischemia and expression of growth factors.

Whether endogenous plasma catestatin also played a role of angiogenesis in the setting of ischemic heart disease, whether plasma catestatin was associated with coronary collateral development, little is known about it. The purpose of the present study was to investigate the associations between endogenous catestatin and coronary collateral grade, and the relationship between catestatin and vascular endothelial growth factor (VEGF) among the patients with chronic myocardial ischemia, especially in the setting of coronary artery CTO.

## Methods

### Subjects

**Fig 1:**
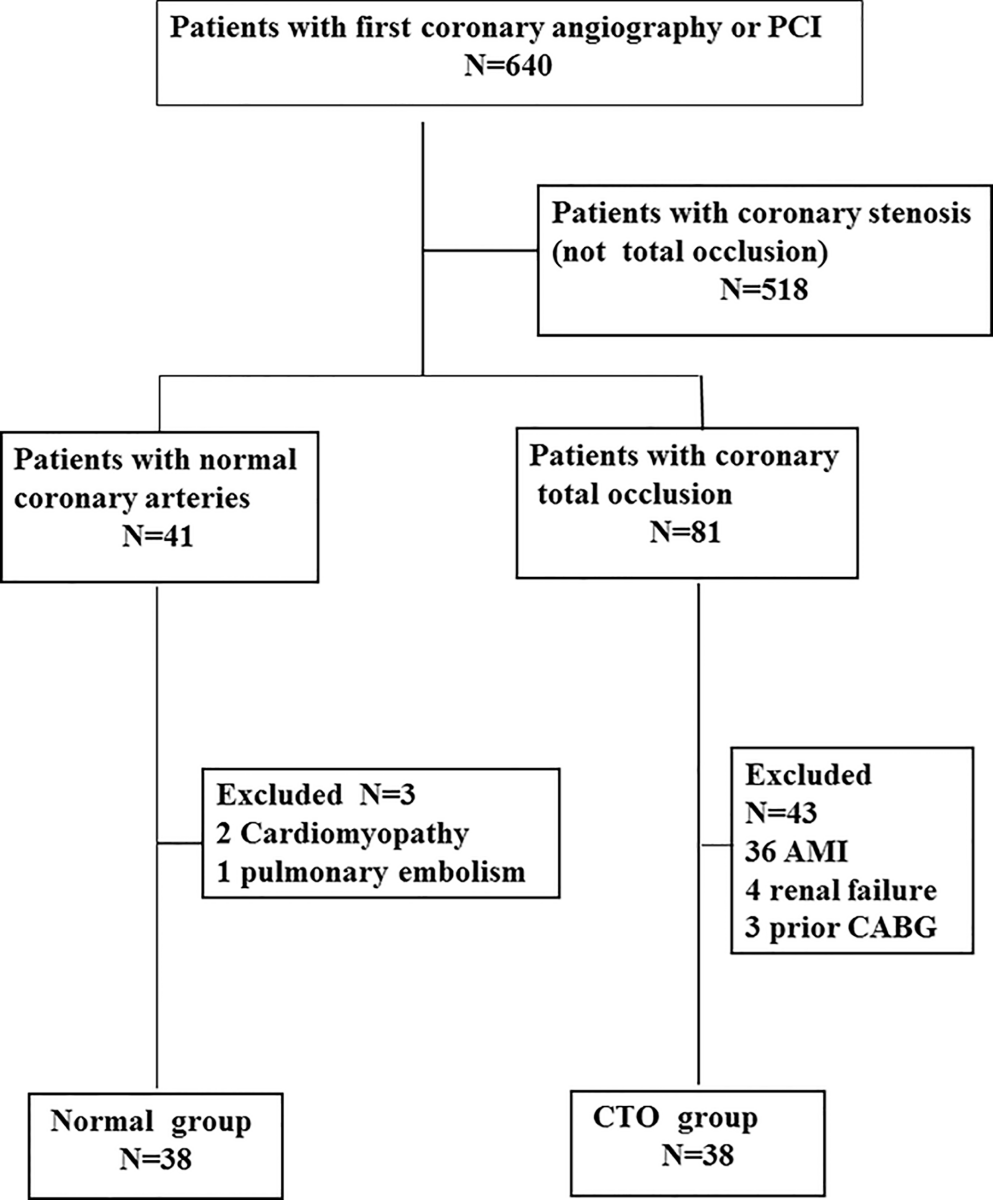
The flowchart of the study subjects. The 640 patients with chest pain for suspicious CAD who underwent coronary angiography or percutaneous coronary intervention (PCI) were screened in series. According to the results of angiography, 518 patients had coronary stenosis (not CTO), 41 patients had normal coronary angigraphy, 81 patients had coronary total occlusion lesions. There were 3 patients with normal coronary angiography and 43 patients with coronary total occlusion were excluded. Finally, there were 38 patients in normal group and 38 patients in CTO group, respectively.

The 640 patients with chest pain for suspicious CAD who underwent coronary angiography or percutaneous coronary intervention (PCI) for the first time from Jan 2008 to Jun 2009 in Peking University Third Hospital were screened in series. According to the results of angiography, 518 patients had coronary stenosis (not CTO), 41 patients had normal coronary, and 81 patients had coronary total occlusion. The patients with CTO or totally normal coronary arteries were enrolled. The following patients were excluded: 1) The patients with acute myocardial infarction within 3 months; 2) The patients with a prior revascularization (percutaneous coronary intervention or coronary artery bypass graft (CABG)); 3) rheumatic heart disease, severe valvular heart disease, cardiomyopathy, 4) deep venous thrombosis or acute pulmonary embolism, infection, systemic inflammatory or tumor disease, chronic pulmonary disease, oxygen saturation less than 95%; 5) documented renal failure or clinical evidence of renal impairment. Finally, 46 patients were excluded (3 patients in normal artery group and 43 patients in CTO group mainly because of myocardial infarction within 3 months);38 patients had at least one coronary chronic total occlusion (CTO) lesion in the major coronary arteries (artery diameter > = 1.5 mm), and 38 patients with normal coronary artery were also enrolled in the series. The details were shown in the Fig 1. The study was conducted in accordance with the Declaration of Helsinki and approved by the Human Subjects Committee of Peking University Third Hospital. All patients provided written informed consent.

### Clinical baseline characteristics

The clinical baseline characteristics, such as age, gender, primary hypertension, diabetes mellitus and hyperlipidemia, body mass index (BMI), smoking, and left ventricular ejection fraction (LVEF) by ultrasound echocardiography, were collected from medical records.

### Coronary angiography and coronary collateral development grading

Coronary angiography were examined by 2 experienced interventional cardiologists who were blind to the study. The standard selective coronary angiograms included at least 4 views of the left coronary system and 2 views of the right coronary artery. The Rentrop score was assessed in standarized manner. Coronary injections was standardized, and collaterals were evaluated at the same time after contrast injection. The details about CTO definition and coronary collateral development grading methods were described in our previous report [21]. CTO was defined as a complete interruption of coronary artery flow lasting more than 3 months. The duration of the occlusion is estimated by evidence on a previous coronary angiography study, a history of MI in territory corresponding to the occluded vessel, or a change in the angina pattern [22]. In the patients with CTO, coronary collateral development was graded according to the Rentrop score method [23]: grade 0, no filling of any collateral vessels; grade 1, filling of side branches of the artery to be perfused by collateral vessels without visualization of epicardial segment; grade 2, partial filling of the epicardial artery by collateral vessels; and grade 3, as complete filling of epicardial artery by collateral vessel. Patients with grade 0–1 collateral development were regarded as poor collateral group and patients with grade 2–3 collateral development were regarded as good collateral group.

### Assays for blood sample

Blood samples were obtained before coronary angiography in all patients. Blood samples were taken into chilled EDTA vacutainers containing 2500 IU/ml aprotinin, then immediately centrifuged at 3000 rpm for 10 min at 4°C, then finally stored at −80°C till analysis. Plasma catestatin level was measured by the catestatin enzyme-linked immunosorbent assay (ELISA) kit (Phoenix Pharmaceuticals, Burlingame, California, USA) according to the manufacturer’s instructions. Plasma VEGF levels were determined by using standard ELISA kits (Human VEGF Immunoassay kit R&D Company, USA).

### Statistical analysis

Continuous variables were given as mean±SD; categorical variables were defined as percentage. Data were tested for normal distribution using the Kolmogorov-Smirnov test. The continuous variables were all normally distributed except age. Age was described using median (25%, 75%) and non-parametric test was used for age comparison. The Student t test or ANOVA test was used for the univariate analysis of the normally distributed continuous variables and the χ^2^ test for the categorical variables. Bivariate correlation analysis (Pearson method) was used for the relationship of two continuous variables. The multiple linear regression was used to evaluate the relationship between catestatin and coronary collateral development. All tests of significance were 2-tailed. Statistical significance was defined as P < 0.05. The Statistical analysis was performed by SPSS statistical software (SPSS 19.0 for Windows; SPSS Inc, Chicago, IL, USA).

## Results

The study population consisted of 38 patients with CTO (CTO group) and 38 with normal coronary arteries (normal group). In the CTO group, 35 patients (92%) had an old myocardial infarction, only 4 patients had a prior angiography. There were 34 patients (89.5%) suffering from multiple vessels, the other 4 patients with single vessel disease. The median time between the old MI and this angiography was 12 months. The baseline characteristics, levels of plasma catestatin of the study population are shown in Table 1. Compared with the normal group, the CTO group was more likely to be older (median 60.5vs 48.5, p = 0.001) and to be smoker (57.9% vs 34.2%, p = 0.038). There were no significant differences in the other baseline characteristics including the medications (p>0.05). The mean levels of plasma catestatin in CTO group were significantly higher than that in normal group (1.97±1.01 vs 1.36±0.97ng/ml, p = 0.009).

**Table 1:** The differences in clinical characteristics and plasma catestatin levels between normal group and CTO group.

Variables	Normal group (n = 38)	CTO group (n = 38)	P value
Age*	48.5(45.8, 59.5)	60.5(51.8, 72.3)	0.001
**Male**	65.8	73.7	0.454
**Hypertension (%)**	71.1	71.1	1.000
**Dyslipidemia (%)**	50.0	50.0	1.000
**Diabetes Mellitus (%)**	13.2	31.6	0.054
**Smoker (%)**	34.2	57.9	0.038
**Medications**			
**for hypertension(%)**	55.3	63.2	0.484
**For dyslipidemia(%)**	36.8	44.7	0.353
**for diabetes(%)**	7.9	21	0.103
**BMI (kg/m^2^)**	26.4±4.1	26.1±3.2	0.821
**LVEF (%)**	67.6±9.9	63.1±10.9	0.091
**Catestatin (ng/ml)**	1.36±0.97	1.97±1.01	0.009

CTO: coronary artery chronic total occlusions; BMI: body mass index; LVEF: left ventricular ejection fraction.

*: median (25%, 75%) of age was shown in the table.

**Fig 2:**
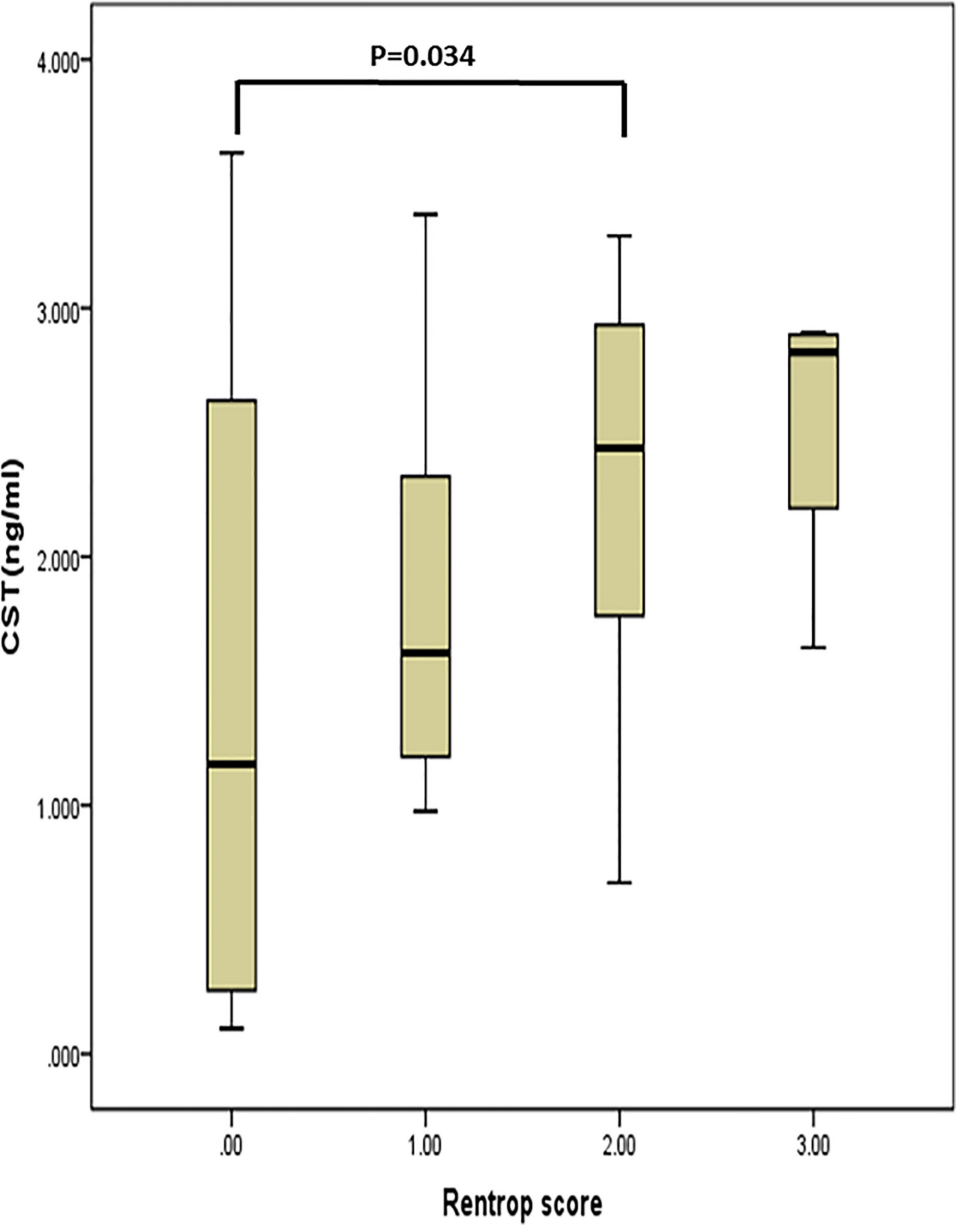
The relationship between plasma catestatin levels and Rentrop scores. The catestatin levels in the different Rentrop score groups were respectively 1.47±1.29 ng/ml (grade0, n = 12); 1.83±0.82 ng/ml (grade1, n = 8); 2.31±0.77 ng/ml (grade2, n = 14); and 2.54±0.61 ng/ml (grade3, n = 4). The differences was significant between grade0 group and grade2 group (p = 0.034).

In the CTO group, the patients were divided into two groups (20 with poor collateral development, and 18 with good collateral development). Table 2 shows the differences between poor and good collateral development groups. The patients with good collateral development was older (median 66.0 vs 51.0, p<0.001), and they had significantly higher catestatin and VEGF levels than those with poor collateral development (2.36±0.73 vs 1.61±1.12 ng/ml, p = 0.018; 425.23±140.10 vs 238.48±101.00pg/mL, p < .001). In the correlation analyses, there is a positive correlation between plasma catestatin levels and Rentrop scores (r = 0.40, p = 0.013) among the patients with CTO. The catestatin levels in different Rentrop score groups were respectively 1.47±1.29 ng/ml (grade0, n = 12); 1.83±0.82 ng/ml (grade1, n = 8); 2.31±0.77 ng/ml (grade2, n = 14); and 2.54±0.61 ng/ml (grade3, n = 4). The differences were significant between grade 0 group and grade 2 group (p = 0.034) (see Fig 2). There is no correlation between plasma catestatin levels and VEGF (r = -0.06, p = 0.744).

**Table 2:** The differences between good collateral group and poor collateral group among CTO patients.

Variables	poor collateral group (n = 20)	good collateral group (n = 18)	P value
Age*	51.0(47.0, 61.3)	66(59.5, 74.3)	<0.001
**Male**	70	77.8	0.587
**Hypertension (%)**	65	77.8	0.386
**Dyslipidemia (%)**	65	33.3	0.051
**Diabetes Mellitus (%)**	45	16.7	0.061
**Smoker (%)**	55	61.1	0.073
**BMI (kg/m^2^)**	25.9±2.6	26.5±4.5	0.738
**LVEF (%)**	65.9±7.9	60.6±12.8	0.164
**Catestatin (ng/ml)**	1.61±1.12	2.36±0.73	0.018
**VEGF(pg/mL)**	238.48±101.00	425.23±140.10	<0.001

CTO: coronary artery chronic total occlusions; BMI: body mass index; LVEF: left ventricular ejection fraction; VEGF: vascular endothelial growth factor.

*: median (25%, 75%) of age was shown in the table.

In the multiple linear regression models, plasma catestatin, age were the independent factors of coronary collateral development after adjustment for gender, hypertension, dyslipidemia, diabetes Mellitus, and smoker (Table 3).

**Table 3:** The associations between plasma catestatin and coronary collateral development by multiple linear regression.

Variables	B	SE	Beta	P value
**Constant**	-0.859	0.814		0.295
**Age**	0.037	0.010	0.449	<0.001
**Gender**	-0.461	0.269	-0.221	0.091
**Hypertension**	-0.011	0.219	-0.005	0.959
**Dyslipidemia**	-0.005	0.202	-0.003	0.980
**Diabetes Mellitus**	-0.427	0.238	-0.186	0.078
**Smoker**	0.064	0.226	0.033	0.777
**Catestatin**	0.247	0.104	0.264	0.020

## Discussion

In the present study, the patients with CTO had higher plasma catestatin than the patients with normal coronary angiography. The plasma catestatin was significantly higher in the patients with better coronary collateral development. The plasma catestatin was one of the independent predictors of coronary collateral development.

To the best of our knowledge, the present study is the first study to investigate the associations between plasma catestatin and collateral development in human with CTO. The patients with good coronary collateral development had higher plasma catestatin, and plasma catestatin level was one of independent predictor for coronary collateral development. Our results were in accordance with the previous study. Theurl M [19] reported that catestatin acts as a novel proangiogenic factor in vitro and in vivo. Catestatin induced migration, proliferation in endothelial cells and exerted capillary tube formation. This effect was via G protein, mitogen-activated protein kinase(MAPK), fibroblast growth factor (bFGF), and bFGF receptor signaling resulting in angiogenesis. To assess the effects of VEGF or bFGF on catestatin-mediated in vitro angiogenesis, catestatin was incubated with neutralizing VEGF and bFGF antibodies. Inhibition of VEGF did not influence catestatin-induced tube formation, but bFGF antibody completely inhibited catestatin-mediated effects. Additionally, bFGF antibody inhibited catestatin-induced MAPK activation in endothelial cells. Furthermore, inhibition of catestatin did not influence VEGF-induced angiogenesis via extracelluar signal-regulated kinase (ERK) activation in endothelial cells. The studies in vivo including the mouse cornea neovascularization assay and the hindlimb ischemia model further demonstrated the angiogenesis role of catestatin, such as increments of blood perfusion and number of capillaries, density of arterioles/arteries and incorporation of endothelial progenitor cells. Our study showed that catestatin was associated with the coronary collateral development, and catestatin was not related with VEGF. The association implies a possible role for catestatin on human angiogenesis.

Recently, Crippa et al. [24] has reported that circulating CgA has dual role of inhibiting angiogenesis in normal conditions, yet accelerating local angiogenesis in damaged tissues, e.g. after cleavage by thrombin. CgA contains a functional anti-angiogenic site in the C-terminal region 410–439 and a latent (or less active) site in the N-terminal region, corresponding to vasostatin-1. Both full-length CgA and vasostatin-1 can inhibit bFGF and VEGF. Full-length CgA contains a latent pro-angiogenic site within residues 352–372 which was corresponding to the catestatin region. Cleavage of CgA at residue R373 by thrombin generates the fragment CgA_1–373_, catestatin, can promote angiogenesis by inducing the release of bFGF from endothelial cells [19, 24]. Therefore, systemically circulating CgA and vasostatin-1 maintain endothelial cell quiescence by their anti-angiogenic potencies. Local activation of thrombin increases CgA_1-373_ at the expense of full-length CgA, shifting the local balance toward a pro-angiogenic state [24–25]. Thus, circulating CgA and CgA-derived polypeptides seemingly form a balance of anti- and pro-angiogenic factors tightly regulated by proteolysis.

As we all know, thrombosis plays a major role in the pathogenesis of the CAD [26]. In the setting of CTO, thrombosis can trigger the circulating CgA system to switch from anti-angiogenic state to pro-angiogenic activity. Therefore, the patients with CTO had higher plasma catestatin. This is an important compensatory mechanisms to myocardial ischemia, because higher catestatin can promote angiogenesis and form better coronary collateral development which can provide an extra blood supply and salvage the myocardium in the ischemic region.

Our study implies that endogenous plasma catestatin could be a potential important biomarker in the patients with CTO. The patients with higher catestatin had better collateral development. In the previous studies, catestatin had an antihypertensive effects [11–13], and catestatin could also improve post-ischemic left ventricular function and decreases ischemia/reperfusion injury [27]. As all the cardioprotective characteristics were taken into account, catestatin might be a potent treatment target for the patients with cardiovascular diseases.

There are some limitations to be discussed. Firstly, the study sample is small. Secondly, the other factors for angiogenesis, such as endothelial progenitor cell (EPC) mobilization, hepatocyte growth factor (HGF), fibroblast growth factor (FGF), insulin-like growth factor (IGF), especially bFGF, were not evaluated. We did not assess the levels of CgA and its other fragments, such as vasostatin [7, 28] which was proved to be an inhibitor of angiogenesis. Thirdly, this was a case-control study, it could not draw a causal relationship. Future studies with larger sample size and better design with more information were needed to assess the potential of catestatin as a biomarker for coronary artery disease and a therapeutic target for collateral development. At last, the age of the CTO can certainly affect the collateral formation, but it is very difficult to determine accurately the age of the CTO. In the CTO group, 35 patients (92%) had an old myocardial infarction. The other 3 patients had no definite history of myocardial infarction. They had stable pectoris, and then suffered a long time chest pain which was longer than 30min and was different with the previous symptom. We guessed that the time of chest pain pattern change was the beginning of CTO. But it is impossible to know exactly at what time the CTO developed for these 3 patients and this is a limitation of the study. Therefore, we excluded the 3 patients without old myocardial infarction and reanalyzed the data, the conclusion did not change.

In conclusion, the plasma catestatin was one of independent predictors of coronary collateral development among the patients with CTO. The plasma catestatin was not related with VEGF. Plasma catestatin may be a useful biomarker for coronary collateral development and potential target for therapeutic angiogenesis in patients with CTO. Further studies are required to clarify this relationship between catestatin and coronary collateral development, the potential mechanisms and the complex interactions of CgA and its fragments.
